# Relationship Between Simple Renal Cysts and Hypertension: A Systematic Review and Meta-Analysis

**DOI:** 10.3390/jcm14165725

**Published:** 2025-08-13

**Authors:** Michael Kitlinski, Harshita Kaushik, Zbigniew Heleniak, Alicja Dębska-Ślizień

**Affiliations:** Department of Nephrology, Transplantology and Internal Medicine, Medical University of Gdansk, 80-210 Gdansk, Poland

**Keywords:** simple renal cyst, Bosniak type 1 cyst, hypertension

## Abstract

**Background**: Simple renal cysts (SRCs) are the most common incidentally found cystic lesions in the kidney. While their association with hypertension (HT) has been explored in various studies, the findings remain inconclusive. Thus, our meta-analysis aimed to systematically evaluate the relationship between SRCs and HT (PROSPERO ID: CRD42025580609). **Methods**: We conducted searches in PubMed, Web of Science Core Collection, and Scopus to identify observational studies that examined the association between SRCs and HT. All articles containing animal or pediatric (<18 years old) study populations or having <10 patients in total and/or lacking a control group that did not develop HT were excluded. Two reviewers independently screened the studies and extracted the data, and the quality of each included study was assessed using the Newcastle–Ottawa Scale. Statistical analyses were performed using Review Manager 5.4. **Results**: In total, 12 studies with 147,310 participants were included in this meta-analysis. Presence of SRCs was associated with a 2.04-fold higher likelihood of having HT (OR 2.04, 95%CI 1.70–2.45, *p* < 0.0001). Multivariate analysis further revealed that SRCs were independently associated with HT (aOR 1.36, 95%CI 1.24–1.49, *p* < 0.0001), with multiple SRCs (aOR 1.36, 95%CI 1.26–2.42, *p* = 0.0008) and bilateral SRCs (aOR 2.26, 95%CI 1.12–4.59, *p* = 0.02) showing a stronger association. **Conclusions**: This study provides the first in-depth review on the topic, showing an established link between SRCs and HT even after adjustment for major confounding factors such as age, sex, renal function, and other metabolic factors.

## 1. Introduction

Simple renal cysts (SRCs), nowadays more commonly referred to as Bosniak type 1 cysts, are fluid-filled benign cysts commonly observed with advanced age [1]. The overall prevalence of SRCs is reported to be 10.7%, with rates ranging from 2.38% in the second decade of life to 35.29% in the seventh decade or later. They are also more commonly observed in males, with a male-to-female ratio of 2.81 [2]. Other factors which may be associated with the presence of SRCs are kidney disease and other cardiovascular diseases [2,3,4]. SRCs are usually asymptomatic as they are incidentally detected with imaging studies of the abdomen [3]. The typical sonographic features on ultrasound of a SRC are as follows: anechoic, thin imperceptible wall, and no internal septations or debris [5].

SRCs have for the longest time been believed to be benign with no serious long-term health consequences. Yet, newly aggregated data over the last decade has been suggestive for a link between SRCs and cardiovascular diseases such as HT and aortic aneurysms [6]. Hypertension (HT) is among the most common risk factors for cardiovascular disease, chronic kidney disease, stroke, and premature mortality worldwide, and is the leading cause of preventable disability [7,8]. Approximately 1.28 billion adults aged 30–79 are affected globally. Despite the significant risks, nearly 46% of individuals with hypertension are unaware of their diagnosis, only 42% receive treatment, and just 20% achieve adequate blood pressure control [9]. Identifying risk factors for hypertension is therefore critical for early detection, timely intervention, and prevention of serious complications.

Several studies have evaluated the association between SRCs and hypertension; however, conclusive evidence remains limited due to inconsistent findings, heterogenous study populations, different definitions for SRCs, and insufficient adjustment for confounding factors such as age, sex, and other comorbidities. To the best of our knowledge, there is no up-to-date review collecting all evidence or meta-analysis concerning this issue. Thus, we sought to perform a systematic review and meta-analysis to comprehensively evaluate the current evidence on the relationship between SRCs and HT.

## 2. Materials and Methods

### 2.1. Search Strategy

The screening process was performed according to the guidelines of Preferred Reporting Items for Systematic Reviews and Meta-Analyses (PRISMA). PubMed, Web of Science Core Collection, and Scopus were used to retrieve studies from inception to 31 August 2024, without language limits. The search term appropriately modified to each database can be found in Table S1. The search was performed independently by two authors (M.K.) and (H.K.) and author (Z.H.) arbitrated any disagreements on inclusion or exclusion of the studies. This study protocol was registered through PROSPERO (ID: CRD42025580609).

### 2.2. Selection Criteria

In the title and abstract screening (1st screening phase), all observational studies in the language of English, involving patients with SRCs and hypertension were evaluated. In the full-text screening (2nd screening phase), it was required for studies to provide extractable data on hypertension and its association with the presence of SRCs and/or their size, number, or localization. All articles containing animal or pediatric (<18 years old) study populations, or having <10 patients in total, and/or lacking a control group that did not develop HT, were excluded.

### 2.3. Quality and Publication Bias Evaluation

The quality of each included study was assessed using the modified Newcastle–Ottawa Scale (NOS). A maximum number of 2 points could be given within the comparability category while in the remaining ones, a maximum of 1 could be given. A total score of (1) ≥7 indicated a study of high quality, (2) 6–4 indicated a study of moderate quality, and (3) ≤3 indicated a low quality study. The quality assessment was performed independently by two authors (M.K. and H.K.) and in case of any disagreements, the concerned study was discussed. Publication bias among the studies was assessed by visual inspection of a funnel plot to search for asymmetry.

### 2.4. Statistical Analysis

Extractable data on hypertension and its association to the presence of SRCs and/or their size, number, distribution, or localization was pooled into an overriding odds ratio (OR) with a 95% confidence interval (Cl). Additionally, studies providing an adjusted odds ratio (aOR) were pooled into a generic inverse model for multivariate analysis. The Z test was performed for overall effect using the Cochran–Mantel–Haenszel method. Subgroup analysis on sample size (>10,000 vs. <10,000 total subjects) or geographical location (European vs. non-European) was performed. Heterogeneity of the overall OR was calculated using the Cochrane test. However, regardless of the degree of heterogeneity, a random effects model was applied in all analyses performed. A *p*-value < 0.05 was considered statistically significant. Forest and funnel plots were generated using RevMan Version 5.4 (Cochrane IMS, Copenhagen, Denmark).

## 3. Results

**Figure 1:**
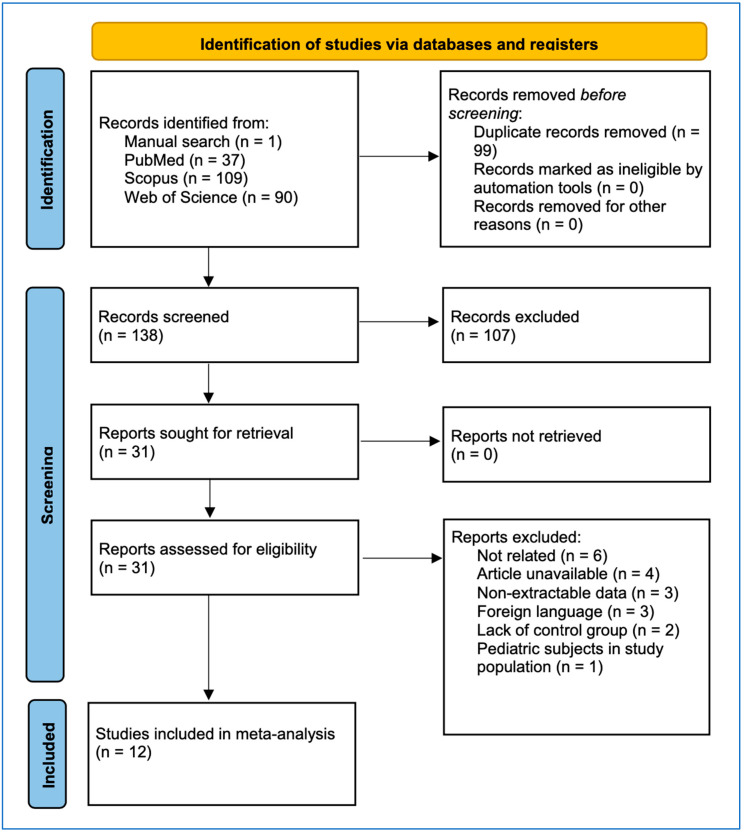
Flowchart of the studies included in the meta-analysis.

A total of 37, 90, and 109 records were identified by using the modified search strategy in PubMed, Web Of Science Core Collection, and Scopus, respectively. Moreover, one record was obtained through manual searches. After removal of duplicates, 138 records remained, which subsequently underwent the first screening phase, leading to an exclusion of 107 records. The remaining 31 records underwent the second screening phase, where six were not related, four were unavailable, three provided no extractable data, three were in a foreign language, two lacked a proper control group, and one record contained pediatric subjects in the study population (Figure 1). Thus, it resulted in a total of 12 studies included in the meta-analysis (Table 1).

After quality assessment of the twelve observational studies with the Newcastle–Ottawa Scale, eight studies were deemed to be of high quality, four of moderate quality, and zero of low quality [10,11,12,13,14,15,16,17,18,19,20,21] (Table S2).

A total amount of twelve studies contained extractable data for univariate analysis on the association between the presence of SRCs and HT. For multivariate analysis, six, four, two, and two studies provided extractable data for the relationship between HT and the presence of SRCs, their number, distribution, and localization, respectively. Age, sex, renal function, and other cardiometabolic factors were some of the confounders adjusted for in the multivariate analyses (Table S3).

**Figure 2:**
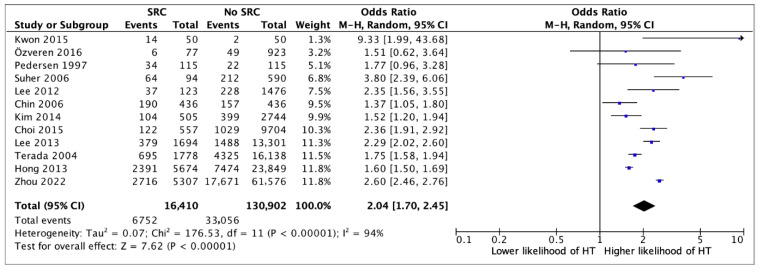
Univariate analysis on the association between the presence of a simple renal cyst (SRC) and hypertension (HT) [10,11,12,13,14,15,16,17,19,20,21].

**Figure 3:**
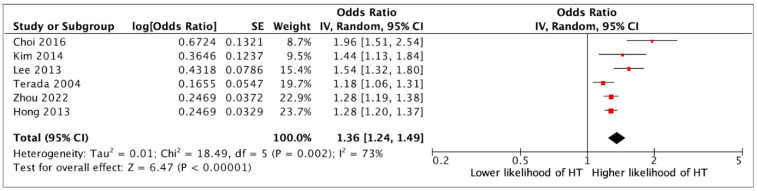
Multivariate analysis on the association between the presence of a simple renal cyst and hypertension (HT) [12,13,14,21].

**Figure 4:**
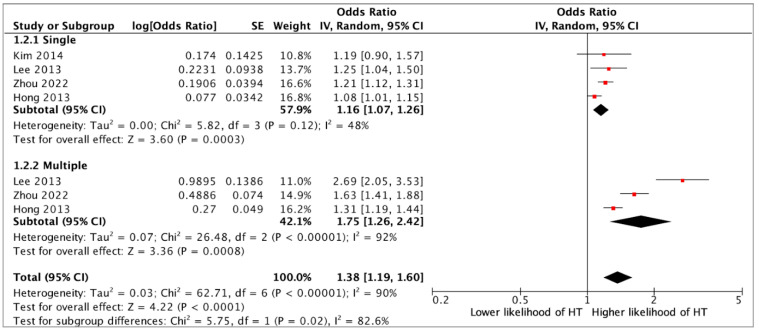
Multivariate analysis on the association between the number of simple renal cysts (single or multiple) and hypertension (HT) [12,13,14,20].

**Figure 5:**
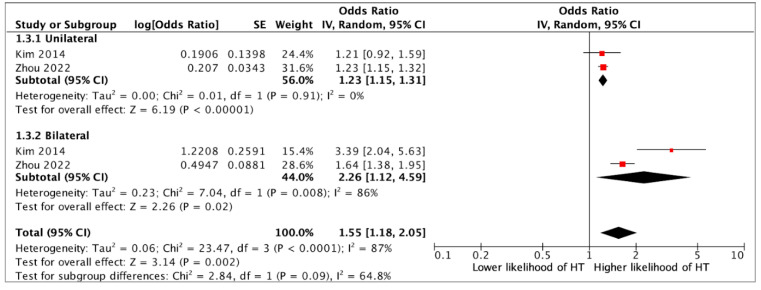
Multivariate analysis on the association between the distribution of simple renal cysts (unilateral or bilateral) and hypertension (HT) [12,20].

**Figure 6:**
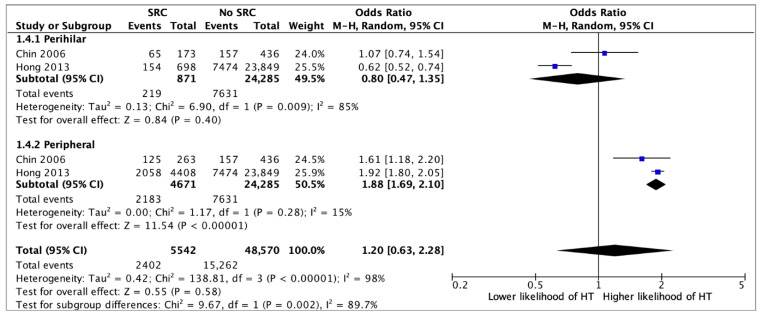
Univariate analysis on the association between the localization (perihilar or peripheral) of simple renal cysts and hypertension (HT) [11,13].

Presence of a SRC was associated with an over two-fold higher likelihood of having HT (OR 2.04, 95%CI 1.70–2.45, *p* < 0.0001) (12 studies) (Figure 2). In multivariate analysis, the association between SRCs and HT remained statistically significant (aOR 1.36, 95%CI 1.24–1.49, *p* < 0.0001) (six studies) (Figure 3). Although a single SRC increased the likelihood of HT (aOR 1.16, 95%CI 1.07–1.26, *p* = 0.003), multiple SRCs were more strongly associated with HT (aOR 1.36, 95%CI 1.26–2.42, *p* = 0.0008) (four studies) (Figure 4). Similarly, SRCs with bilateral distribution had as stronger association with HT (aOR 2.26, 95%CI 1.12–4.59, *p* = 0.02) (two studies) (Figure 5) than those with unilateral distribution (aOR 1.23, 95%CI 1.15–1.31, *p* < 0.00001) (two studies) (Figure 5). Moreover, SRCs with peripheral localization were associated with HT (OR 1.88, 95%CI 1.69–2.10, *p* = *p* < 0.00001), while those in perihilar were not (OR 0.8, 95%CI 0.47–1.35, *p* = 0.4) (two studies) (Figure 6). Subgroup analysis based on geographical location (European vs. non-European) and size of study population for each study included (>10,000 vs. ≤10,000) was consistent, and thus unsuccessful in explaining the high heterogeneity observed (Supplementary Figures S1–S3).

The funnel plots exhibited an asymmetrical distribution in the graph between the OR and SE (LogOR), suggesting a chance of publication bias (Supplementary Figures S4 and S5).

## 4. Discussion

To the best of our knowledge, this is the first systematic review and meta-analysis on SRCs and their association with HT. With a study population of over 140,000 patients derived from a total of 12 observational studies, our meta-analysis found SRCs to be associated with HT, independent of confounding factors such as age, sex, renal function, or other cardiometabolic disturbances. Both SRCs and HT are prevalent conditions in the general population, and the establishment of such a positive relationship raises the clinical question of whether patients with SRCs and concomitant HT would benefit from a more individualized treatment approach for their HT.

SRCs are largely considered to be acquired, with their number and size known to increase with age [2,16,22]. SRCs have also been associated with male sex, renal dysfunction, and smoking [2,21]. Accordingly, SRCs and HT may coexist due to shared etiologic factors. This is reflected by our finding for a stronger association in univariate analysis when compared to multivariate analysis. However, after adjustment for the above-mentioned confounding factors in the pooled multivariate analysis, we still found SRCs to be independently associated with HT—suggesting a true relationship which is still to be fully understood.

It is speculated that renal ischemia and injury prompts an aberrant hypertrophic response leading to cyst growth and subsequently nephron loss due to compensatory hyperfiltration [10,23,24], which in turn would lead to secondary activation of the renin–angiotensin–aldosterone system (RAAS) and an increase in blood pressure (BP). On the other hand, HT may be potentially caused directly by the presence of a SRC, by its compression of the renal arteries also triggering an activation of RAAS [25]. This theory is supported by the observation of normalized BP in patients with HT after renal cystectomy or percutaneous decompression of SRCs [12,20,25,26,27]. SRCs located in the perihilar region are in closer proximity to renal vessels and thus should be more likely associated with RAAS activation and increased BP. Not supporting this theory is our finding that SRCs with peripheral locations were associated with HT, while those in perihilar locations were not. As this observation was seen in a univariate analysis, it very likely could be due to differences in characteristics of SRCs depending on their locations. SRCs located in the peripheral region tend to be larger and more often multiple as compared to those located in the perihilar region [11], and thus could indirectly bring a higher predisposition for expanding onto renal structures. Moreover, as we also found in this study, bilateral and multiple SRCs exhibited the highest association with HT, which could be related to a more serious and widespread SRC expansion on the healthy renal parenchyma, causing a greater activation of the RAAS. Yet, non-RAAS dependent mechanisms linking SRCs to HT need to be considered, as the association between the conditions was also found to be independent of serum renin levels [20]. Structural weakness of arterial and renal cyst walls, attributable to impairment of the extracellular matrix (ECM), could contribute to this connection. Multiple studies have demonstrated elevated levels of matrix metalloproteinases (MMP) in renal cysts [28,29], suggesting a role in disrupting ECM integrity and promoting cyst formation. Elevated MMPs have similarly been implicated in vascular remodeling processes that facilitate the development and progression of HT [30].

The prognostic impact of HT in the setting of SRCs is uncertain, as it is for the most part unexplored. Lu et al. found SRCs to be associated with a poorer prognosis in subjects with type B aortic dissections and HT [31]. Average ambulatory BP measurements seem also to be higher in those with SRCs as compared to non-SRC subjects, and presence of SRCs was found to be independently associated with a non-dipping BP phenomenon [32]. As earlier mentioned, there is a potential for curative management of HT by treating the SRC, yet further data is required to test its feasibility on a large scale. However, another aspect is that those with SRCs might benefit by adhering to a more individualized pharmacotherapy, as reports suggest that certain antihypertensive drugs (e.g., calcium channel blockers) might have more risk than benefit, as they might further exacerbate the growth of the cyst [33].

Our study is subject to a handful of limitations. Firstly, it may be argued that a “true” SRC in order to be considered absolutely benign should fulfill the radiographic features of a Type 1 renal cyst according to the Bosniak classification [34]. Although many of the included studies defined SRCs based on typical features for benign lesions (such as lack of calcifications, echolucent, and a thin wall), only two studies (16.6%) mention defining SRCs directly based on the Bosniak classification. Yet, the majority of SRCs in clinical setting will be only be ever visualized using ultrasound. Despite our study identifying an association between SRCs and HT independent of other risk factors, our study design is unable to answer or draw conclusions on which condition came first, and whether treatment of SRC may safely and cost-effectively alleviate or even cure HT. Thus, future studies evaluating the issue of whether SRCs are a modifiable risk factor for HT are warranted. Lastly, although the overall evidence is of moderate–high quality, our findings need to be interpreted cautiously with regards to the study population. As the majority of the included studies consisted of subjects from Asian ethnicities and are of a retrospective nature, this remains a major limitation amidst the current literature. Therefore, the independent association between SRCs and HT can currently only be applied to this geographical area, and further population-based studies with Western study populations are necessary to confirm these findings. Additionally, a modest amount of heterogeneity was observed in the analyses even after subgroup analyses to explore its root. We believe the observed heterogeneity to be mainly related to the inconsistent use of various definitions for SRCs and HT.

## 5. Future Directions

Future prospective studies on SRCs and their association with hypertension should adopt a standardized definition of SRCs as Bosniak type I cysts, to ensure consistency across studies. Suggested confounding factors like smoking [2], age, sex, renal function, and a history of antihypertensive treatment and other cardiovascular disease(s) [6] could help establish a cause–effect relationship and answer the question of which event truly causes the other. Additionally, randomized controlled trials comparing interventional approaches like renal cystectomy and decompression of cysts with non-interventional management would also aid enormously in establishing causality as well as assessing the clinical benefits of treating SRCs with regards to changes in BP and renal function.

In conclusion, independent of risk factors such as sex, age, renal disease, or other cardiometabolic disturbances, our meta-analysis observed consistent evidence for SRCs to be associated with an increased likelihood of having HT. From a clinical point of view, patients with SRCs would benefit from closer monitoring of blood pressure, and studies on whether this group of subjects would benefit from a more personalized treatment of HT remain warranted.

## Figures and Tables

**Table 1:** Characteristics of the included studies.

Study	Country	Study Design	Level of Quality (NOS)	Mean Age (SD)	Definition for HT	Definition for SRC	Explicit Exclusion of Subjects with Polycystic Kidney Disease and/or Other Cystic Kidney Disease	SRC	No SRC	Total
Pedersen 1997 [10]	Denmark	Retrospective cohort	High	67.7 (31–91)	Diastolic BP ≥ 95 mm Hg	Cysts ≥ 10 mm, a round anechoic lesion that lay at least partly within the renal contour and showed distal echo enhancement	No	115	115	230
Chin 2006 [11]	South Korea	Retrospective cohort	High	50.7 (m) and 50.9 (f)	NR	Anechoic lesion with homogeneity, water content, and sharp interface to renal parenchyma that did not have thickening, calcifications, or enhancement.	Yes	436	436	872
Zhou 2022 [12]	China	Retrospective cohort	High	46.36 ± 13.35	BP ≥ 140 and or/90 or controlled BP due to use of an antihypertensive drug	Cystic mass in or on the surface of the kidney, with no echo, a clear boundary, good internal sound transmission, enhanced posterior echo, a wall that is slender and hyperechoic, an inner wall that is smooth, and a spherical or ellipsoid morphology.	Yes	5307	61,576	66,883
Hong 2013 [13]	South Korea	Retrospective cohort	High	56.03 ± 8.675 (SRC), 52.28 ± 7.933 (NSRC)	BP ≥ 130/85 or the use of antihypertensive drugs	Lack of internal echoes, and a smooth, sharply defined wall which was not suggestive of malignancy.	Yes	5674	23,849	29,523
Ting Lee 2013 [14]	Taiwan	Retrospective cohort	High	NR	BP ≥ 140/90 or documented history of HT	Echolucent, round or oval, thin-walled, smooth contours, well defined, sharply demarcated posterior wall, no calcifications, no doppler signals from within the cyst, and no sound wave amplification behind the cyst, which was not suggestive of a malignancy.	Yes	1694	13,301	14,995
Özveren 2016 [15]	Turkey	Retrospective cohort	High	42.76 ± 10.89	Systolic BP > 140, diastolic BP > 90, or use of antihypertensive drugs	Type 1 renal cyst according to the Bosniak classification.	No	77	923	1000
Choi 2016 [16]	South Korea	Retrospective cohort	Medium	52.67 ± 0.31 (SRC) and 46.82 ± 0.09 (NSRC)	Systolic BP > 140/and or diastolic BP > 90, use of antihypertensive drugs	Absent internal echoes, smooth sharply defined walls, and posterior acoustic enhancement, indicating posterior through-transmission that was not suspicious for a renal malignancy.	Yes	557	9704	10,261
Suher 2006 [17]	Turkey	Retrospective cohort	Medium	67.3 ± 12.1 years (SRC)	NR	Lack of internal echoes, thin wall, and distal enhancement.	Yes	94	590	684
Lee 2012 [18]	South Korea	Retrospective cohort	High	42.3 ± 6.6 (SRC) and 42.2 ± 6.8 (NSRC)	Systolic BP > 140 and/or diastolic BP > 90, use of antihypertensive drugs	NR	Yes	123	1476	1599
Kwon 2016 [19]	South Korea	Prospective cohort	Medium	59.1 (SRC) and 39.2 (NSRC)	Mean blood pressure ≥ 130/85 mmHg or antihypertensive drug use	Unilateral cysts ≥ 4 cm, water density, homogenous, hairline thin wall, no septa, no calcifications, and no enhancement.	No	50	50	100
Kim 2014 [20]	South Korea	Retrospective cohort	High	54.0 ± 3.6	Systolic BP > 140 or diastolic BP > 90 mmHg, or current use of antihypertensive medications	Internally anechoic, sharply defined, smooth-walled, and round or oval cyst.	No	505	2744	3249
Terada 2004 [21]	Japan	Retrospective cohort	Medium	49.3	Systolic BP > 140 or diastolic BP > 90 mmHg, or current use of antihypertensive medications	Lack of internal echoes and a smooth, sharply defined wall that was not suggestive of malignancy.	No	1778	16,138	17,914

BP = Blood pressure; NR = Not reported; NOS = Newcastle Ottawa Scale; SRC = Simple renal cyst.

## Data Availability

The original contributions presented in this study are included in the article/Supplementary Materials. Further inquiries can be directed to the corresponding author.

## Supplementary Materials

The following supporting information can be downloaded at: https://www.mdpi.com/article/10.3390/jcm14165725/s1, Table S1: Search term strategy appropriately modified to each database, Table S2: Quality of included studies according to the Newcastle–Ottawa Scale, Table S3: Confounding factors (in each individual study) adjusted for in the multivariate analyses, Figure S1: Subgroup analysis (European vs. non-European) on the univariate association between the presence of a simple renal cyst (SRC) and hypertension (HT), Figure S2: Subgroup analysis (study population > 10,000 vs. <= 10,000) on the univariate association between the presence of a simple renal cyst (SRC) and hypertension (HT), Figure S3: Subgroup analysis (study population > 10,000 vs. <= 10,000) on the multivariate association between the presence of a simple renal cyst (SRC) and hypertension (HT), Figure S4: Funnel plot to (asses publication bias) from the univariate analysis on the association between simple renal cysts and hypertension, Figure S5: Funnel plot to (asses publication bias) from the multivariate analysis on the association between simple renal cysts and hypertension.

## References

[1] Carrim Z.I., Murchison J.T. (2003). The Prevalence of Simple Renal and Hepatic Cysts Detected by Spiral Computed Tomography. Clin. Radiol..

[2] Chang C.-C., Kuo J.-Y., Chan W.-L., Chen K.-K., Chang L.S. (2007). Prevalence and Clinical Characteristics of Simple Renal Cyst. J. Chin. Med. Assoc. JCMA.

[3] Garfield K., Leslie S.W. (2024). Simple Renal Cyst. StatPearls.

[4] Ziganshin B.A., Theodoropoulos P., Salloum M.N., Zaza K.J., Tranquilli M., Mojibian H.R., Dahl N.K., Fang H., Rizzo J.A., Elefteriades J.A. (2016). Simple Renal Cysts as Markers of Thoracic Aortic Disease. J. Am. Heart Assoc..

[5] Gimpel C., Avni E.F., Breysem L., Burgmaier K., Caroli A., Cetiner M., Haffner D., Hartung E.A., Franke D., König J. (2019). Imaging of Kidney Cysts and Cystic Kidney Diseases in Children: An International Working Group Consensus Statement. Radiology.

[6] Chewcharat A., Hamaya R., Thongprayoon C., Cato L.D., Mao M.A., Cheungpasitporn W. (2020). The Association between Simple Renal Cyst and Aortic Diseases: A Systematic Review and Meta-Analysis of Observational Studies. J. Evid.-Based Med..

[7] Wu C.-Y., Hu H.-Y., Chou Y.-J., Huang N., Chou Y.-C., Li C.-P. (2015). High Blood Pressure and All-Cause and Cardiovascular Disease Mortalities in Community-Dwelling Older Adults. Medicine.

[8] Forouzanfar M.H., Afshin A., Alexander L.T., Anderson H.R., Bhutta Z.A., Biryukov S., Brauer M., Burnett R., Cercy K., Charlson F.J. (2016). Global, Regional, and National Comparative Risk Assessment of 79 Behavioural, Environmental and Occupational, and Metabolic Risks or Clusters of Risks, 1990–2015: A Systematic Analysis for the Global Burden of Disease Study 2015. Lancet.

[9] World Hypertension Day—17 May 2025. https://eso-stroke.org/blog-world-hypertension-day-17-may-2025/.

[10] Pedersen J.F., Emamian S.A., Nielsen M.B. (1997). Significant Association between Simple Renal Cysts and Arterial Blood Pressure. Br. J. Urol..

[11] Chin H.J., Ro H., Lee H.J., Na K.Y., Chae D.-W. (2006). The Clinical Significances of Simple Renal Cyst: Is It Related to Hypertension or Renal Dysfunction?. Kidney Int..

[12] Zhou Y., Jia L., Lu B., Bai L., Cui W. (2022). Simple Renal Cyst as an Independent Risk Factor for Hypertension. J. Clin. Hypertens..

[13] Hong S., Lim J.H., Jeong I.G., Choe J., Kim C.-S., Hong J.H. (2013). What Association Exists between Hypertension and Simple Renal Cyst in a Screened Population?. J. Hum. Hypertens..

[14] Lee C.-T., Yang Y.-C., Wu J.-S., Chang Y.-F., Huang Y.-H., Lu F.-H., Chang C.-J. (2013). Multiple and Large Simple Renal Cysts Are Associated with Prehypertension and Hypertension. Kidney Int..

[15] Özveren B., Onganer E., Türkeri L.N. (2016). Simple Renal Cysts: Prevalence, Associated Risk Factors and Follow-Up in a Health Screening Cohort. Urol. J..

[16] Choi J.D. (2016). Clinical characteristics and long-term observation of simple renal cysts in a healthy Korean population. Int. Urol. Nephrol..

[17] Suher M., Koc E., Bayrak G. (2006). Simple Renal Cyst Prevalence in Internal Medicine Department and Concomitant Diseases. Ren. Fail..

[18] Lee Y.-J., Kim M.S., Cho S., Kim S.R. (2012). Association between Simple Renal Cysts and Development of Hypertension in Healthy Middle-Aged Men. J. Hypertens..

[19] Kwon T., Lim B., You D., Hong B., Hong J.H., Kim C., Jeong I.G. (2016). Simple Renal Cyst and Renal Dysfunction: A Pilot Study Using Dimercaptosuccinic Acid Renal Scan. Nephrology.

[20] Kim S.-M., Chung T.-H., Oh M.-S., Kwon S.-G., Bae S.-J. (2014). Relationship of Simple Renal Cyst to Hypertension. Korean J. Fam. Med..

[21] Terada N., Arai Y., Kinukawa N., Yoshimura K., Terai A. (2004). Risk Factors for Renal Cysts. BJU Int..

[22] Park H., Kim C.-S. (2015). Natural 10-Year History of Simple Renal Cysts. Korean J. Urol..

[23] Kanbay M., Copur S., Bakir C.N., Covic A., Ortiz A., Tuttle K.R. (2024). Glomerular Hyperfiltration as a Therapeutic Target for CKD. Nephrol. Dial. Transplant. Off. Publ. Eur. Dial. Transpl. Assoc.-Eur. Ren. Assoc..

[24] Floege J., Johnson R.J., Feehally J. (2010). Comprehensive Clinical Nephrology E-Book.

[25] Lüscher T.F., Wanner C., Siegenthaler W., Vetter W. (1986). Simple Renal Cyst and Hypertension: Cause or Coincidence?. Clin. Nephrol..

[26] Aloui S., Bouraoui S., Salem R., Toffahi M., Skhiri H., Frih A., Dhia N.B., Elmay M. (2011). Remission of Arterial Hypertension after the Treatment of a Giant Renal Cyst. Saudi J. Kidney Dis. Transplant..

[27] Pejcic T., Hadzi-Djokic J., Markovic B., Naumovic R. (2011). Resolving Erythrocytosis and Hypertension after Open Surgical Extirpation of Giant Renal Cyst Measuring 30 Cm: Case Report. Ren. Fail..

[28] Obermüller N., Morente N., Kränzlin B., Gretz N., Witzgall R. (2001). A Possible Role for Metalloproteinases in Renal Cyst Development. Am. J. Physiol. Renal Physiol..

[29] Kim E.K., Choi E.R., Song B.G., Jang S.Y., Ko S.M., Choi S.-H., Sung J., Sung K., Choe Y.H., Oh J.K. (2011). Presence of Simple Renal Cysts Is Associated with Increased Risk of Aortic Dissection: A Common Manifestation of Connective Tissue Degeneration?. Heart.

[30] Taherkhani S., Sheibani M., Mohammadkhanizadeh A., Virag J.A.I., de Castro Braz L., Azizi Y. (2025). Metalloproteinases (MMPs) in Hypertensive Disorders: Role, Function, Pharmacology, and Potential Strategies to Mitigate Pathophysiological Changes. Front. Pharmacol..

[31] Lu N., Hu P., Wang J., Yan W., He Z., Xu T., Yu M., Chen S., Ma X., Tan X. (2019). Simple Renal Cysts Are Associated With 24-Month Prognosis of Patients With Type B Aortic Dissection and Hypertension. Can. J. Cardiol..

[32] Afsar B., Afsar R.E., Sen S.T., Kirkpantur A., Eyileten T., Yilmaz M.I., Caglar K. (2011). Simple Renal Cysts and Circadian Blood Pressure: Are They Related to Each Other in Patients with Hypertension?. Int. Urol. Nephrol..

[33] Schiavone C., Salvatore L., Primavera A., Cuccurullo F., Verna N., Di Stefano F., Thomson E., Tenaglia R., Di Gioacchino M. (2003). Simple Renal Cysts in Hypertensive Patients: Relation between Cyst Growing and Anti-Hypertensive Therapy. Int. J. Immunopathol. Pharmacol..

[34] How Simple Are ‘Simple Renal Cysts’?|Nephrology Dialysis Transplantation|Oxford Academic. https://academic.oup.com/ndt/article-abstract/29/suppl_4/iv106/1908338?redirectedFrom=fulltext.

