# Supplementary material for: Relationship Between Simple Renal Cysts and Hypertension: A Systematic Review and Meta-Analysis
Source: J Clin Med. 2025 Aug 13;14(16):5725. doi: 10.3390/jcm14165725 (PMC12387922; doi:10.3390/jcm14165725)
Supplement: Supplementary file 1 [file jcm-14-05725-s001.zip › jcm-3747301-supplementary.pdf]

Table S1. Search term strategy appropriately modified to each database.

1. **PubMed**

#1	"simple kidney cyst"[All Fields] OR "simple renal cyst"[All Fields]	272
#2	"hypertense"[All Fields] OR "hypertension"[MeSH Terms] OR "hypertension"[All Fields] OR "hypertension's"[All Fields] OR "hypertensions"[All Fields] OR "hypertensive"[All Fields] OR "hypertensive's"[All Fields] OR "hypertensives"[All Fields] OR "blood pressure"[MeSH Terms] OR ("blood"[All Fields] AND "pressure"[All Fields]) OR "blood pressure"[All Fields] OR "blood pressure determination"[MeSH Terms] OR ("blood"[All Fields] AND "pressure"[All Fields] AND "determination"[All Fields]) OR "blood pressure determination"[All Fields] OR "arterial pressure"[MeSH Terms] OR ("arterial"[All Fields] AND "pressure"[All Fields]) OR "arterial pressure"[All Fields]	1,138,371
#3	("infant"[MeSH Terms] OR "child"[MeSH Terms] OR "adolescent"[MeSH Terms]) NOT ("adult"[MeSH Terms] OR "aged"[MeSH Terms])	2,216,836
#4	"case reports"[Publication Type]	2,428,485
#5	"animals"[MeSH Terms] NOT "humans"[MeSH Terms]	5,253,071
#6	#1 AND #2 NOT #3 NOT #4 NOT #5	37

2. **Web of Science Core Collection**

#1	TS=((simple_renal_cyst) OR (simple_kidney_cyst))	509
#2	TS=((hypertensi*) OR (blood_pressure))	904,083
#3	TI = ("Case Series" OR "case report")	336,609
#4	#1 AND #2 NOT #3	90

3. **Scopus**

#1	TITLE-ABS-KEY (simple_renal_cyst OR simple_kidney_cyst)	684
#2	TITLE-ABS-KEY ((hypertensi*) OR (blood_pressure))	1,507,342
#3	TITLE ("Case Series" OR "case report")	459,973
#4	#1 AND #2 AND NOT #3	109

Table S2. Quality of included studies according to the Newcastle–Ottowa Scale.

	Selection				Comparability		Exposure		
	Representativeness of the exposed cohort	Selection of the non-exposed cohort	Ascertainment of exposure	Demonstration that outcome of interest was not present at the start of the study	Adjustment for age	Adjustment for other factors (e.g., sex, renal function, cardiovascular disease, obesity, etc.)	Assessment of outcome	Was follow-up long enough for outcomes to occur (**hypertension found at the**	Adequacy of follow up of cohorts (loss-to-follow-up **<20** % or no	NOS Score (out of 9)

									**simultaneous or nearby visit)**	statement)	
Zhou 2022	Retrospective cohort	1	1	1	0	1	1	1	1	0	7
Hong 2013	Retrospective cohort	1	1	1	0	1	1	1	1	0	7
Lee 2013	Retrospective cohort	1	1	1	0	1	1	1	1	0	7
Özveren 2016	Retrospective cohort	1	1	1	0	1	1	1	1	0	7
Choi 2016	Retrospective cohort	1	1	1	0	1	1	0	1	0	6
Suher 2006	Retrospective cohort	1	1	1	0	0	0	1	1	0	5
Lee 2012	Retrospective cohort	0	1	1	1	1	1	1	1	0	7
Kwon 2016	Prospective cohort	1	0	1	0	0	0	1	1	0	4
Kim 2014	Retrospective cohort	1	1	1	1	1	1	1	1	0	8
Terada 2004	Retrospective cohort	1	1	1	0	1	1	0	1	0	6

	Selection				Comparability		Exposure		
	Is the case definition adequate?	Representativeness of the cases	Selection of Controls	Definition of Controls	Adjustment for age	Adjustment for other factors (e.g., sex, renal function, obesity, cardiovascular disease, etc.)	Ascertainment of exposure	Same method of ascertainment for cases and controls	Non-Response rate

Pedersen 1997	Case-control	1	1	1	1	1	0	1	1	0	7
Chin 2006	Case-control	1	1	1	1	1	1	1	1	0	8

Table S3. Confounding factors (in each individual study) adjusted for in the multivariate analyses.

Study (Last name, year):	Factors adjusted for in the multivariate analysis:
Choi 2016	Age, sex, BMI, SBP, DBP, total cholesterol, TG, serum glucose, serum creatinine, serum uric acid, proteinuria, microscopic hematuria, eGFR, DM, hypercholesterolemia.
Kim 2014	Age, BMI, DM, dyslipidemia, alcohol intake, smoking, exercise.
Lee 2013	Age, gender, BMI, DM, eGFR, hypercholesterolemia (>=200 mg/dL), low HDL-cholesterol (male <40 and female <50 mg/dL), regular exercise, smoking, family history of hypertension.
Terada 2004	Age, sex, serum creatinine.
Zhou 2022	Sex, age group, BMI group, heart rate group, eGFR group, smoking, drinking, history of different diseases [diabetes, hyperlipidemia, cardiovascular disease, cerebrovascular disease], family history of HT, renal stone, adrenal change, renal calcification, renal hamartoma, renal hydronephrosis, and nephropathy
Hong 2013	Age, sex, BMI, triglycerides, fasting glucose, eGFR, and uric acid.

BMI = Body-mass-index; SBP = Systolic blood pressure; DBP = Diastolic blood pressure; eGFR = estimated glomerular filtration rate; DM = Diabetes mellitus; HT = Hypertension.

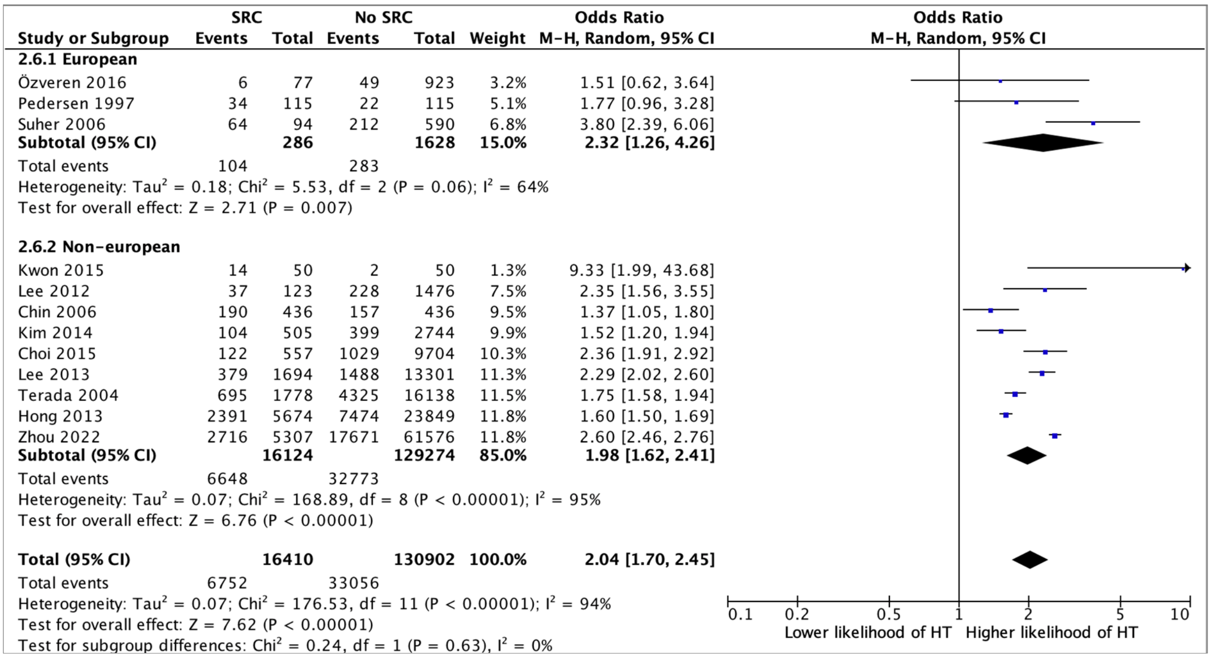

Figure S1. Subgroup analysis (European vs. non-European) on the univariate association between the presence of a simple renal cyst (SRC) and hypertension (HT).

Study or Subgroup	SRC Events	SRC Total	No SRC Events	No SRC Total	Weight	Odds Ratio M-H, Random, 95% CI
**3.6.1 > 10 000**						
Choi 2015	122	557	1029	9704	10.3%	2.36 [1.91, 2.92]
Lee 2013	379	1694	1488	13301	11.3%	2.29 [2.02, 2.60]
Terada 2004	695	1778	4325	16138	11.5%	1.75 [1.58, 1.94]
Hong 2013	2391	5674	7474	23849	11.8%	1.60 [1.50, 1.69]
Zhou 2022	2716	5307	17671	61576	11.8%	2.60 [2.46, 2.76]
**Subtotal (95% CI)**		**15010**		**124568**	**56.8%**	**2.08 [1.63, 2.65]**
Total events	6303		31987			
Heterogeneity: Tau² = 0.07; Chi² = 151.08, df = 4 (P < 0.00001); I² = 97%						
Test for overall effect: Z = 5.94 (P < 0.00001)						
**3.6.2 <= 10 000**						
Kwon 2015	14	50	2	50	1.3%	9.33 [1.99, 43.68]
Özveren 2016	6	77	49	923	3.2%	1.51 [0.62, 3.64]
Pedersen 1997	34	115	22	115	5.1%	1.77 [0.96, 3.28]
Suher 2006	64	94	212	590	6.8%	3.80 [2.39, 6.06]
Lee 2012	37	123	228	1476	7.5%	2.35 [1.56, 3.55]
Chin 2006	190	436	157	436	9.5%	1.37 [1.05, 1.80]
Kim 2014	104	505	399	2744	9.9%	1.52 [1.20, 1.94]
**Subtotal (95% CI)**		**1400**		**6334**	**43.2%**	**2.03 [1.46, 2.83]**
Total events	449		1069			
Heterogeneity: Tau² = 0.12; Chi² = 21.69, df = 6 (P = 0.001); I² = 72%						
Test for overall effect: Z = 4.21 (P < 0.0001)						
**Total (95% CI)**		**16410**		**130902**	**100.0%**	**2.04 [1.70, 2.45]**
Total events	6752		33056			
Heterogeneity: Tau² = 0.07; Chi² = 176.53, df = 11 (P < 0.00001); I² = 94%						
Test for overall effect: Z = 7.62 (P < 0.00001)						
Test for subgroup differences: Chi² = 0.01, df = 1 (P = 0.92), I² = 0%						

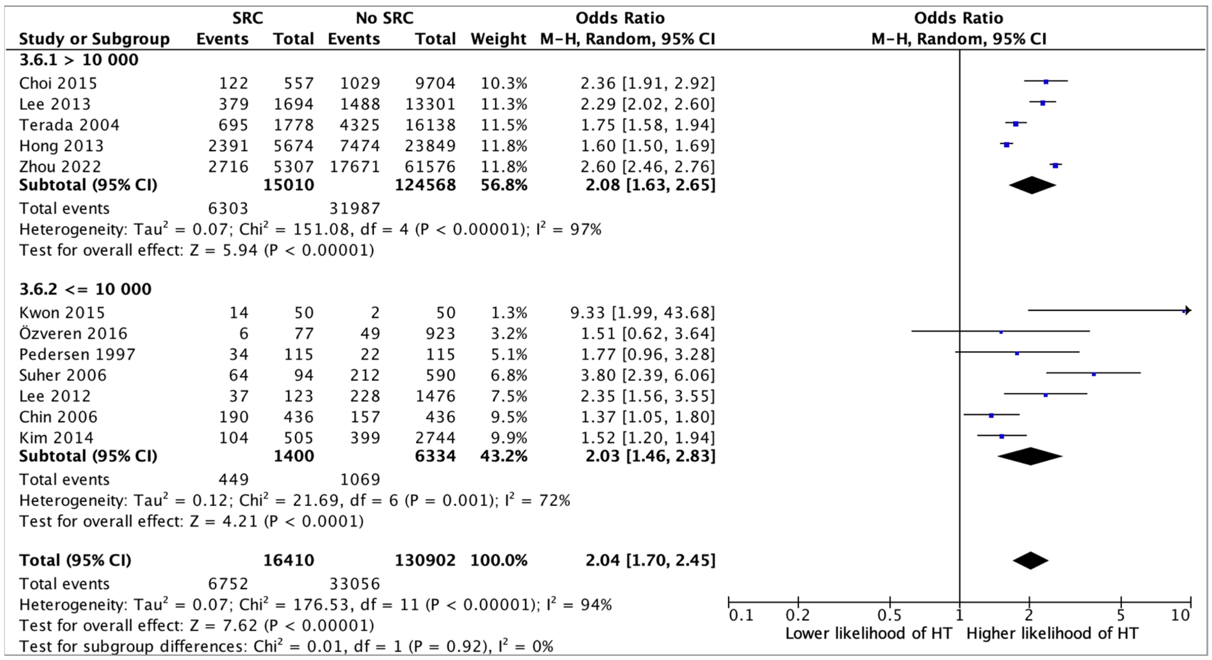

Figure S2. Subgroup analysis (study population > 10 000 vs. <= 10 000) on the univariate association between the presence of a simple renal cyst (SRC) and hypertension (HT).

Study or Subgroup	log[Odds Ratio]	SE	Weight	Odds Ratio IV, Random, 95% CI
**3.1.1 > 10 000**				
Choi 2015	0.6724	0.1321	8.7%	1.96 [1.51, 2.54]
Lee 2013	0.4318	0.0786	15.4%	1.54 [1.32, 1.80]
Terada 2004	0.1655	0.0547	19.7%	1.18 [1.06, 1.31]
Zhou 2022	0.2469	0.0372	22.9%	1.28 [1.19, 1.38]
Hong 2013	0.2469	0.0329	23.7%	1.28 [1.20, 1.37]
**Subtotal (95% CI)**			**90.5%**	**1.35 [1.22, 1.50]**
Heterogeneity: Tau² = 0.01; Chi² = 17.79, df = 4 (P = 0.001); I² = 78%				
Test for overall effect: Z = 5.90 (P < 0.00001)				
**3.1.2 <= 10 000**				
Kim 2014	0.3646	0.1237	9.5%	1.44 [1.13, 1.84]
**Subtotal (95% CI)**			**9.5%**	**1.44 [1.13, 1.84]**
Heterogeneity: Not applicable				
Test for overall effect: Z = 2.95 (P = 0.003)				
**Total (95% CI)**			**100.0%**	**1.36 [1.24, 1.49]**
Heterogeneity: Tau² = 0.01; Chi² = 18.49, df = 5 (P = 0.002); I² = 73%				
Test for overall effect: Z = 6.47 (P < 0.00001)				
Test for subgroup differences: Chi² = 0.21, df = 1 (P = 0.65), I² = 0%				

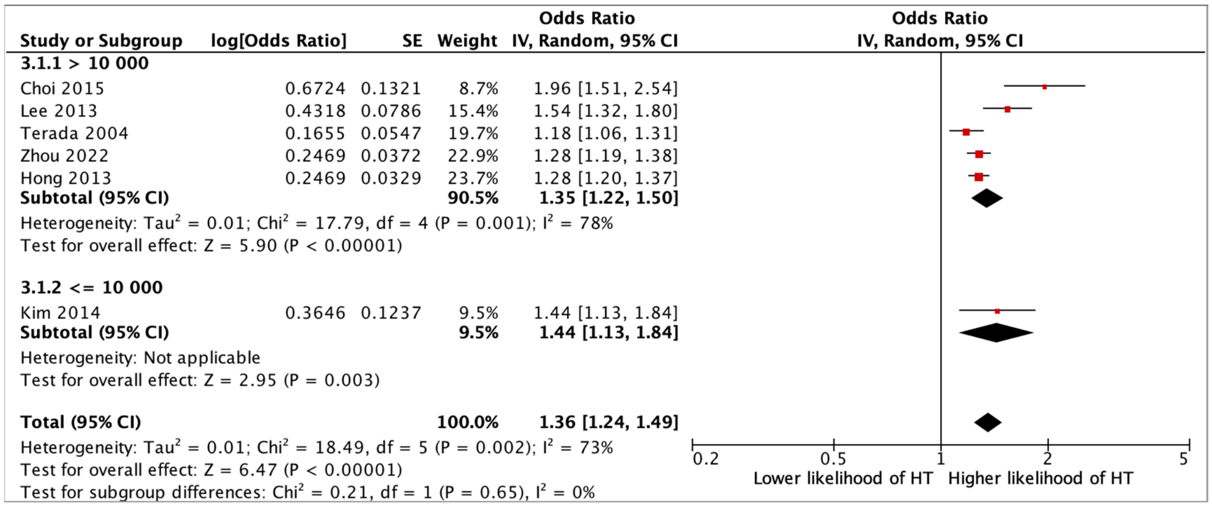

Figure S3. Subgroup analysis (study population > 10 000 vs. <= 10 000) on the multivariate association between the presence of a simple renal cyst (SRC) and hypertension (HT).

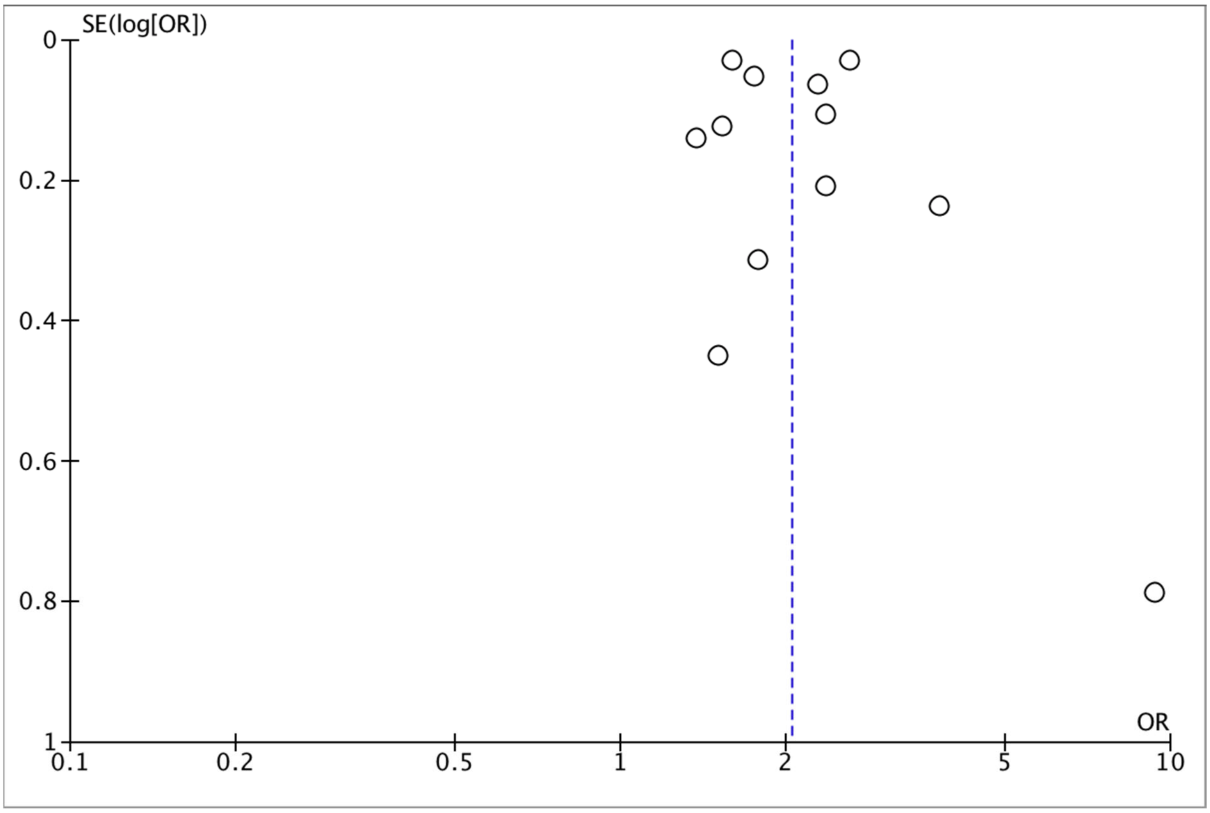

Figure S4. Funnel plot to (asses publication bias) from the univariate analysis on the association between simple renal cysts and hypertension.

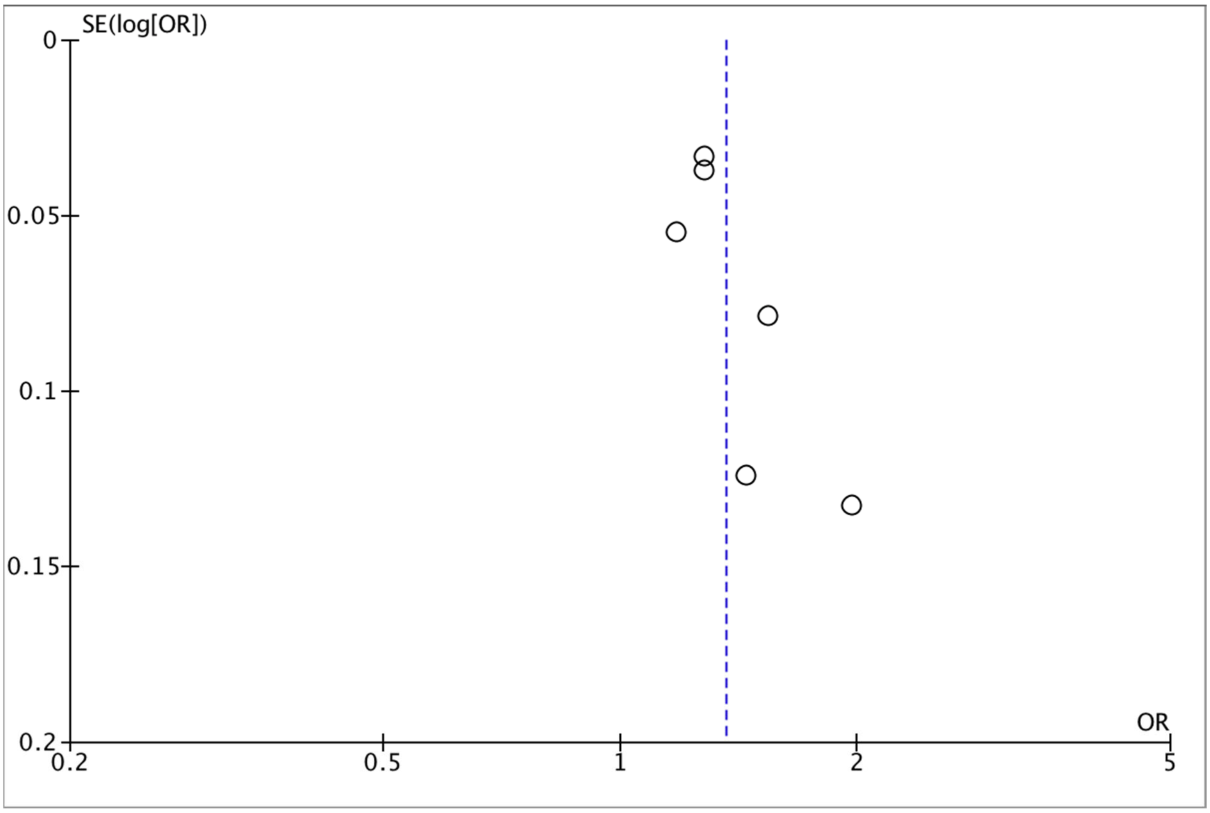

Figure S5. Funnel plot to (asses publication bias) from the multivariate analysis on the association between simple renal cysts and hypertension.
